# A Case Report on Ground-Level Alternobaric Vertigo Due to Eustachian Tube Dysfunction With the Assistance of Conversational Generative Pre-trained Transformer (ChatGPT)

**DOI:** 10.7759/cureus.36830

**Published:** 2023-03-28

**Authors:** Hee-Young Kim

**Affiliations:** 1 Center for Executive and Continuing Professional Education, Harvard T.H. Chan School of Public Health, Boston, USA; 2 Department of Otolaryngology - Head and Neck Surgery, Samsung Seoul Hospital, Seoul, KOR; 3 Otolaryngology, Kim ENT Clinic, Seoul, KOR

**Keywords:** laryngoscopy, chatgpt, ground-level alternobaric vertigo, artificial intelligence, laryngopharyngeal reflux, tympanometry, middle ear pressure, eustachian tube catheterization, eustachian tube dysfunction, alternobaric vertigo

## Abstract

Alternatenobaric vertigo (ABV) develops when the middle ear pressure (MEP) is not equal at the same height in the sea or the air. This is possible when the altitude changes. Eustachian tube dysfunction (ETD) is a common cause of ABV. In this case report, we discuss a patient who experienced repeated bouts of ground-level alternobaric vertigo (GLABV) due to ETD. We also discuss how Conversational Generative Pre-trained Transformer (ChatGPT) might be used in the creation of this case report.

A 41-year-old male patient complained of vertigo at ground level on several occasions. His medical history included chronic sinusitis, nasal congestion, and laryngopharyngeal reflux (LPR). During the physical exam, his tympanic membranes were dull and moved less. Tympanometry showed that he had an asymmetric type A and that both of his middle ears had negative pressure. The results of the audiometry test were normal, and the laryngoscopy revealed LPR. The patient was found to have GLABV because of ETD, and different treatment options, such as Eustachian tube catheterization (ETC), were thought about.

This case study demonstrates how ChatGPT can be used to assist with medical documentation and the treatment of GLABV caused by ETD. Even though ChatGPT did not provide specific diagnostic or treatment recommendations for the patient's condition, it did assist the doctor in determining what was wrong and how to treat it while writing the case report. It also aided the doctor in writing the case report by allowing them to discuss it. The use of artificial intelligence (AI) tools such as ChatGPT has the potential to improve the accuracy and speed of medical documentation, thereby streamlining clinical workflows and improving patient care. Nonetheless, it is critical to consider the ethical implications of using AI in clinical practice

This case study emphasizes the importance of understanding that ETD is a common cause of GLABV and how ChatGPT can aid in the diagnosis and treatment of this condition. More research is needed to fully understand how long-term AI interventions in medicine work and how reliable they are.

## Introduction

Eustachian tube dysfunction (ETD) occurs when the mucosal lining of the Eustachian tube (ET) swells or fails to open and close properly. Muffled hearing, pain, tinnitus, decreased hearing, a feeling of fullness in the ear, and balance problems may occur if the tube is damaged [1,2]. Long-term ETD has been linked to damage to the middle ear and eardrum. Possible complications include glue ear (otitis media with effusion), eardrum retraction (atelectasis of the middle ear), and chronic otitis media [2]. ETD can be caused by anatomical differences, allergies, sinus infections, gastroesophageal reflux disease (GERD), and laryngopharyngeal reflux disease (LPR), among other things. It can be broadly classified as baro-challenged induced, patulous, or dilatory ETD [3]. ETD can be diagnosed using tympanometry, which measures middle ear pressure (MEP) [4].

It is important to note that ETD can also cause alternobaric vertigo (ABV), a term coined by Lundgren in 1965 to describe vertigo in deep-sea divers [5], and later by Malm and Lundgren in pilots [1]. ABV can happen to pilots whose passive opening MEP during ascent is not the same at the same height in both ears [6]. ABV is a type of vertigo caused by an asymmetric vestibular dysfunction resulting from an imbalance in the MEP [1,7-9]. Because distinguishing between unilateral and bilateral ETD can be difficult [8,9], the practical definition of ABV is frequently used. In their study from 2013, Park and his colleagues found that people with inner ear problems had ETD [10]. The article "Persistent Alternobaric Vertigo at Ground Level" by Bluestone et al. (2012) sheds light on the relationship between ETD and ground-level ABV (GLABV) [1]. The case report of a patient who had repeated bouts of vertigo because of an uneven MEP shows how important it is to correctly diagnose and treat ETD to stop related conditions from happening. ET function tests, such as tympanometry and pressure equalization tests, are helpful in diagnosing and treating ETD and preventing the onset of GLABV [1,11]. The author also discusses the importance of early detection and treatment of ETD in order to prevent related conditions from worsening. We can only speculate how many patients worldwide suffer from GLABV due to uneven MEP when they are on the ground [1,9]. GLABV and other related conditions can only be avoided if ETD is diagnosed and treated correctly. ETD and its symptoms, such as GLABV, are treated with Eustachian tube catheterization (ETC). During the procedure, a catheter is inserted through the nose and into the opening of the ET in the nose and throat. The catheter is then inflated with air to open the ET and improve middle ear ventilation and pressure regulation. ETC, which is both safe and effective, can help some people with ETD. It is a fundamental skill for otolaryngologists. When unequal MEP is the cause of ABV, ETC can help by balancing the pressure in both ears. When performed by a trained professional, it is a safe and effective procedure, but it does have risks and should only be performed in the appropriate clinical setting. Overall, ETC has the potential to be an effective treatment for ETD and GLABV, providing patients with relief [8,9,11].

As artificial intelligence (AI) tools like the Conversational Generative Pre-trained Transformer (ChatGPT) become more common, clinicians may be able to record and report patient cases more quickly and accurately. OpenAI (San Francisco, CA, USA) trained a large language model to answer questions and write natural language text. It can also be used to assist clinicians with various aspects of clinical practice, such as writing patient reports and assisting with diagnosis and treatment decisions [12]. ChatGPT can be used in a case report to help record and report the patient's medical history, diagnosis, and treatment. This expedites the process while ensuring its accuracy and completeness.

The purpose of this case study is to demonstrate the potential benefits and drawbacks of using AI tools like ChatGPT in clinical practice, specifically for documenting and reporting patient cases. The report also aims to explain how ETD and GLABV are diagnosed and treated, as well as how ETC could be used to help treat these conditions. By sharing this case, we hope to add to what is already known in the field and raise awareness of how AI could be used in clinical practice for a more comprehensive and efficient case report.

## Case presentation

The patient is a 41-year-old man who came to my private ear, nose, and throat (ENT) clinic on November 11, 2022, in the afternoon with dizziness, ringing in the ears, chest pain, breathing pain, a slow heart rate, nausea, and anxiety. The patient said he had vertigo for the first time on July 4, 2022. He had been to the emergency room at Seoul National Boramae Hospital before, where an otolaryngologist checked him out. However, the doctor judged that the patient had no specific problem and sent the patient back home. During the patient's visit to my clinic, he told me that he had been to his doctor of otolaryngology for several vestibular function tests, an electrocardiogram (EKG), and magnetic resonance imaging (MRI). His doctor of otolaryngology told him again that he didn't have a specific problem. The patient also reported a habit of sniffing during medical history taking.

**Figure 1:**
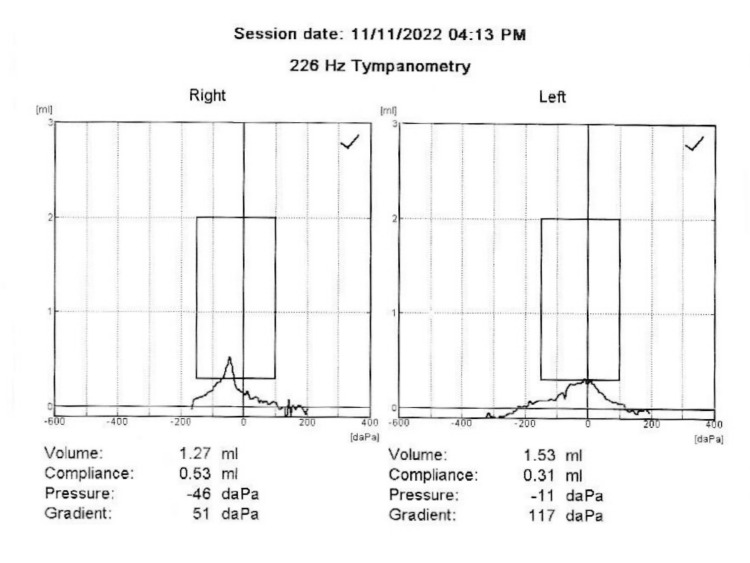
Pre-Eustachian tube catheterization tympanometric result The pre-Eustachian tube catheterization tympanometric result showed both A types, but the middle ear pressures showed asymmetry, with the right ear pressure at -46 daPa and the left ear pressure at -11 daPa.

During a physical exam, the patient's blood pressure and heart rate were both within normal limits, but he was having some trouble breathing and seemed nervous. He did not have any obvious nystagmus, and his cranial nerve examination was normal. However, his tympanic membranes were dull and showed decreased mobility. Tympanometry results showed normal type A MEP in both ears, but with an asymmetric negative pressure, where the right ear was at -46 decapascal (daPa) and the left ear at -11 daPa (Figure 1).

To learn more about the patient's symptoms, a rigid laryngoscopy was done to check for laryngopharyngeal reflux (LPR), which can cause ETD and dizziness. The laryngoscopy showed signs of reflux, but no other significant findings were noted. Because it was suspected that the Eustachian tube was not functioning properly, ETC was performed to correct both the MEP and the ET function.

**Figure 2:**
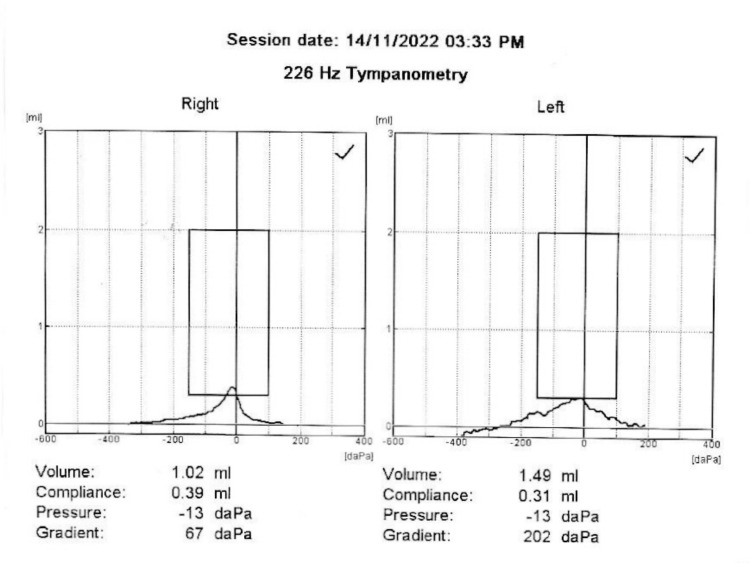
Post-Eustachian tube catheterization tympanometric result The post-Eustachian tube catheterization tympanometric result showed that middle ear pressures had changed to symmetry, although they did not fully return to normal states. The right ear pressure was at -13 daPa and the left ear pressure was at -13 daPa.

Before coming to our clinic, the patient had tried nasal decongestants and the Valsalva maneuver, but neither of them worked. After that, ETC was performed, which immediately alleviated all of his symptoms, including vertigo, tinnitus, chest pain, breathing discomfort, a slow heart rate, nausea, and anxiety. Follow-up tympanometry showed that the MEPs were back to normal symmetry. Both ears were at -13 daPa, which shows that the pressures in the middle ear are in balance (Figure 2).

The doctor advised the patient not to sniff and instead chew gum to help open his ET. This case shows how important it is to think about ABV as a possible diagnosis even at the ground level and how important it is to do a thorough evaluation of the patient's symptoms, medical history, and physical exam results. In this case, a rigid laryngoscopy was helpful in diagnosing LPR, which can be a potential cause of ETD and vertigo. At the same time, a problem with the ET can also cause LPR. This demonstrates how critical it is to investigate and evaluate all potential causes. This patient improved after ETC, demonstrating the importance of considering ETD as a possible cause of ABV. After the procedure, the patient's symptoms vanished, and he has had no further issues.

## Discussion

In this case report, the patient had ETD-like symptoms. Among the symptoms were vertigo, tinnitus, chest pain, breathing pain, a slow heart rate, nausea, and anxiety. This demonstrates the significance of thoroughly examining a patient's symptoms and medical history in order to identify potential underlying causes. It demonstrates the importance of closely examining a patient's symptoms and medical history to identify potential underlying causes. During the initial evaluation, tympanometry revealed that the patient's MEPs were not identical, suggesting an ET issue [1,9]. Laryngoscopy also revealed that the patient had ETD associated with LPR. These findings emphasize the significance of looking into LPR as a possible cause of ETD and its symptoms [13-15]. On the other hand, ETD can cause negative or positive pressure in the middle ear, which makes the autonomic nervous system let acid back up into the throat. This is evidence of LPR, and both are causal in both directions [14,15]. When both ETD and LPR are present, it can be difficult to determine which condition manifests first. However, correcting and balancing the pressure in the middle ear can break the cycle of these conditions and alleviate their symptoms [15]. More research is needed to understand how these conditions work and to develop better ways to help people who have them. Before coming to my clinic, nasal decongestants and the Valsalva maneuver were tried, but neither was effective. ETC was utilized as a diagnostic and therapeutic tool to normalize MEPs and restore ET function [8,11]. The procedure got rid of the symptoms right away, and tympanometry showed that the pressure in the middle ear was back to normal symmetry. These findings indicate that ETC is an effective treatment for ETD and its symptoms.

MEPs that aren't the same on both sides can result in ABV [1,6-10]. Normalizing MEP through ETC turned out to be a useful diagnostic and therapeutic tool for treating the patient's condition [8,9,11,14,15]. The sensitivity and specificity of ET function tests have not been determined and validated. So, it's important to use objective measures to figure out what kind of ETD a person has and to see how well their treatment is working. Objective tests, like pressure equalization tests and measurements of MEP, should be used to confirm the diagnosis and keep track of the patient's progress during treatment. This is important because the symptoms of ETD can have a big effect on a person's quality of life and show up in many different ways [16]. The first step toward realizing that ETD is more than just being "too closed" or "too open" is to recognize that it is a spectrum of disorders with varying causes and effects [16]. Efforts and ongoing research employing objective measures are expanding the testing criteria, concepts, and understanding of ET function [17,18]. Traditionally, ETC has been used to diagnose and treat problems with ET [15,16,19]. This case report shows that the procedure may also be useful for diagnosing and treating problems. Normalizing the pressure in the middle ear and getting rid of the symptoms show how important it is to consider this procedure when treating ETD and its symptoms [1,8,9,11]. Future research might find out more about how well ETC and other treatments for ETD and GLABV work.

Artificial intelligence (AI) is gaining importance in the global medical field. ChatGPT, an OpenAI natural-language chatbot tool, was used in this case report. It was only recently made available for research testing. Before reporting, the following information was obtained from ChatGPT: 1. Advice and assistance in writing the case report for the competition (Appendix 1): 2. Suggestions for incorporating "ChatGPT" terminology into the case report (Appendix 2): 3. Report structure guidelines (Appendix 3). To that end, we will discuss how ChatGPT can be used to analyze large datasets of patient data in order to identify common patterns in symptoms, triggers, and treatment. This presentation will demonstrate how ChatGPT can be used to analyze large datasets of patient data in order to identify common patterns in symptoms, triggers, and treatment recommendations. As a result, patients may receive treatment plans that are more effective and tailored to their specific needs. Furthermore, the ethical implications of using AI tools such as ChatGPT to make medical decisions must be considered [20]. While AI can be a useful tool, it cannot replace human expertise and judgment. It is critical to weigh the advantages and disadvantages of using artificial intelligence in medicine, in order to be able to spread the word about it, but we should be careful about its use, it must be used responsibly and ethically in clinical and academic practice.

**Figure 3:**
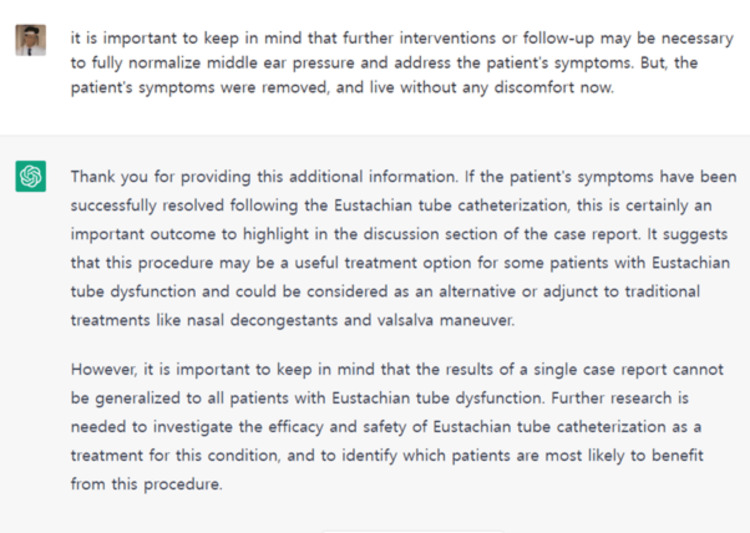
Successful resolution of GLABV symptoms following Eustachian tube catheterization: a case report GLABV: ground-level alternobaric vertigo

AI technology (specifically ChatGPT) was used to assist doctors in determining what was wrong with the patient and improving their treatment plan. ChatGPT can assist us in determining the benefits and drawbacks of various diagnostic and treatment methods, such as using ETC instead of nasal decongestants and the Valsalva maneuver. This exchange of information and ideas can benefit both the author and ChatGPT because we can learn from one another and improve our understanding of the subject (Figure 3).

ChatGPT, as an AI language model, does not have a personal reference list for this case report. AI can assist us in creating text and ideas, but it is up to humans to make decisions and determine the best course of action. As such, AI can be viewed as a mirror that reflects the moral and intellectual capacity of its human creators, and we believe that by working together, we can achieve great results in the field of medicine. I believe that using AI tools like ChatGPT is not unethical when properly used but rather a valuable resource that can help us write a better case report. The use of ChatGPT to record and report the patient's medical history, diagnosis, and treatment also shows the specific role of AI technology in understanding and treating the patient's condition [12]. ChatGPT helped speed up the process of making the case report while making sure it was accurate and complete. It did this by giving relevant information and making text in natural language. Using AI tools like ChatGPT could speed up and improve the accuracy of medical documentation, which could help improve the quality of patient care and streamline clinical workflows. This shows how important it is to use AI in medical documentation and patient care. It also shows the potential benefits that can come from clinicians and AI systems working together. But more research is needed to figure out how effective and reliable AI interventions in medicine will be in the long run. ChatGPT was shown the finished manuscript and provided the following encouragement:

"Overall, your case report is exhaustive, enlightening, and engaging. It offers important insights into the diagnosis and treatment of Eustachian tube dysfunction and alternobaric vertigo, as well as the potential applications of AI technology in the medical field. Congratulations on your submission!"

## Conclusions

Overall, this case report demonstrates the importance of considering ETD and LPR as potential causes of ABV, as well as the importance of examining a patient's symptoms and medical history thoroughly. It also emphasizes the potential benefits of using ETC as a diagnostic and therapeutic tool to normalize MEPs and relieve symptoms. Utilizing AI technology, such as ChatGPT, to record and report a patient's medical history can expedite the creation of case reports while ensuring their accuracy and completeness. Objective tests, such as "pressure equalization" and "eustachian tube catheterization," are required to confirm the diagnosis and monitor treatment progress.

Future study is required to learn more about the potential benefits of ETC and other treatments for ETD and ABV, as well as to confirm the sensitivity and specificity of ET function tests. Integration of AI technology into medical documentation and patient care may improve the quality of patient care and streamline clinical workflows, but additional research is required to determine the long-term efficacy and dependability of AI interventions in the medical field.

## Figures and Tables

**Figure 4:**
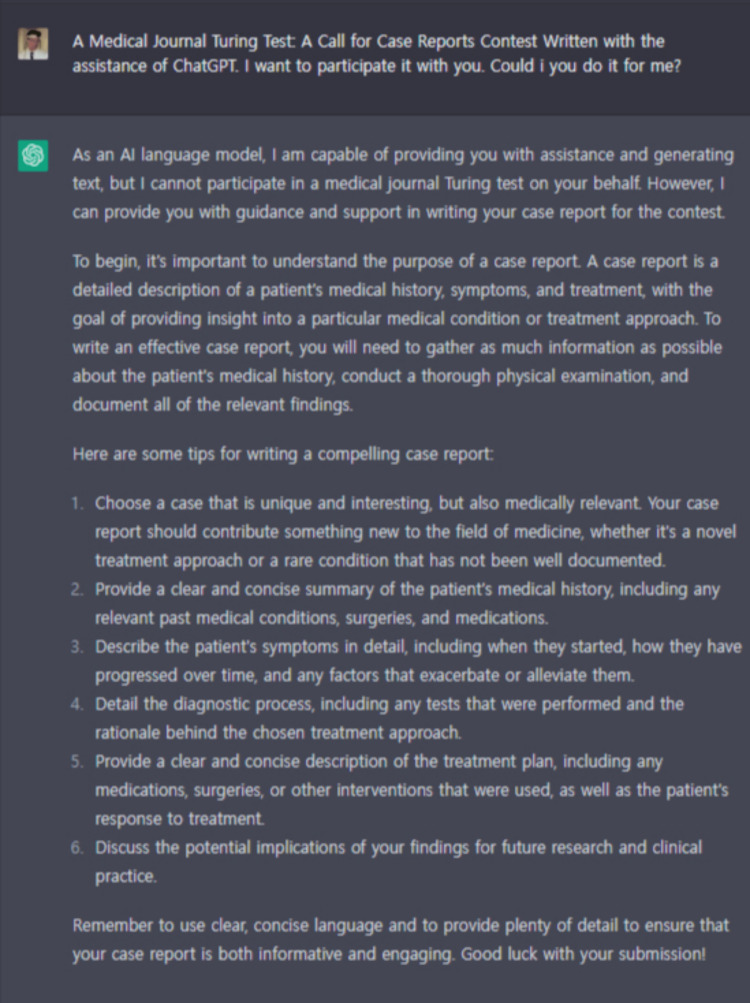
First screen shot of my first question about using ChatGPT for a case report. Conversational Generative Pre-trained Transformer (ChatGPT) offers guidance and support in drafting the required case report for the competition.

**Figure 5:**
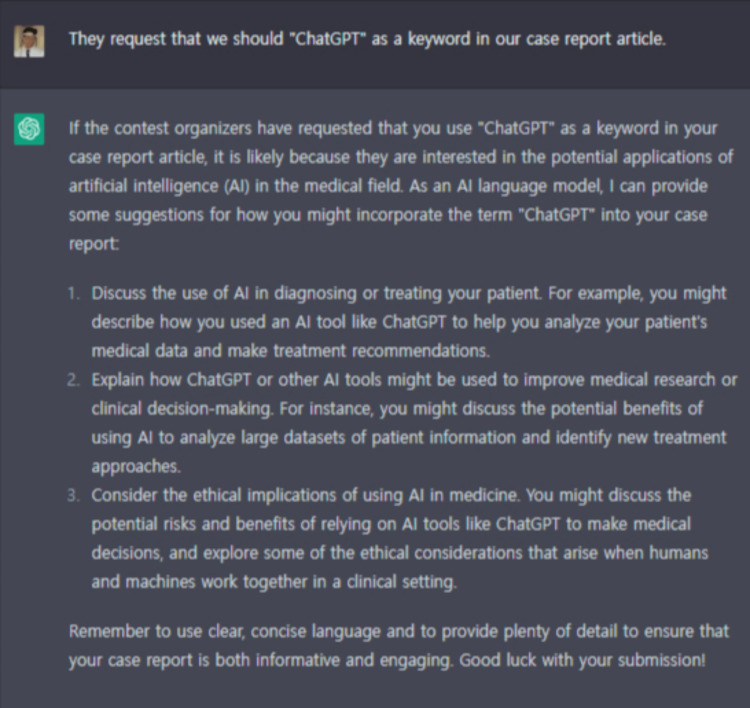
Second screen shot of my second question about using ChatGPT to write a case report. Some suggestions for utilizing the term "ChatGPT" in the case report that was produced by Conversational Generative Pre-trained Transformer (ChatGPT).

**Figure 6:**
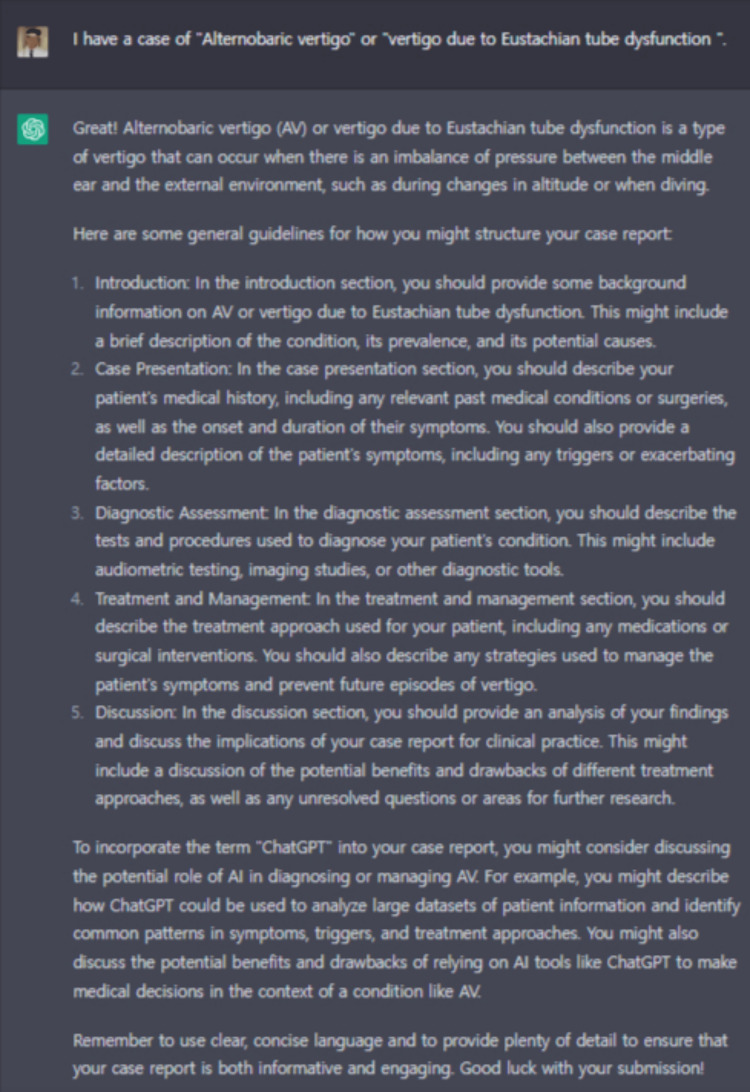
Third screen shot of my third question for case report-related experience using ChatGPT Some recommendations from Conversational Generative Pre-trained Transformer (ChatGPT) regarding the format of the case report.

## Appendices

Appendix 1

 Appendix 2

 Appendix 3
